# How to optimise breast cancer staging with contrast-enhanced mammography: current evidence and clinical implications

**DOI:** 10.1186/s13244-026-02331-3

**Published:** 2026-06-29

**Authors:** Chiara Bellini, Giulia Bicchierai, Diego de Benedetto, Federica di Naro, Chiara Maiello, Sofia Vidali, Paolina Tonelli, Ermanno Vanzi, Cecilia Boeri, Vittorio Miele, Jacopo Nori

**Affiliations:** 1 Breast Imaging Unit, Department of Radiology, Azienda Ospedaliero-Universitaria Careggi, Florence, Italy; 2 Department of Radiology, Azienda Ospedaliero-Universitaria Careggi, Florence, Italy; 3 Department of Experimental and Clinical Biomedical Sciences “Mario Serio”, University of Florence, Florence, Italy

**Keywords:** Breast neoplasms, Mammography, Contrast media, Preoperative care

## Abstract

**Abstract:**

Contrast-enhanced mammography (CEM) is a functional breast imaging technique that combines conventional mammographic morphology with contrast-based assessment of tumour vascularity. In the presurgical setting, CEM has gained interest as a tool to improve local staging by refining tumour extent evaluation and detecting additional disease beyond conventional imaging. This narrative review critically summarises current evidence on the role of CEM in preoperative breast cancer staging. Technical aspects, diagnostic performance for index lesion size assessment and detection of additional malignant foci, comparison with breast MRI, and impact on surgical planning are discussed, with attention to histological subtypes and clinically relevant scenarios encountered in daily practice. Available studies suggest that CEM provides tumour extent assessment comparable to MRI in selected settings, with higher specificity for additional lesions and meaningful influence on surgical decision-making. However, limitations related to contrast administration, radiation dose, evaluation of posterior structures, and nodal staging remain. Heterogeneity in protocols and outcome definitions also limits generalizability. CEM represents a valuable adjunct for presurgical breast cancer staging when appropriately integrated into multimodality workflows. Prospective multicenter studies are needed to define its impact on surgical outcomes and clarify its role within personalised breast cancer management.

**Critical Relevance Statement:**

This review provides a critical overview of CEM in preoperative breast cancer staging, highlighting its role as a complementary imaging tool when MRI is unavailable or unsuitable, and emphasising the need for prospective multicentre studies to validate its clinical impact.

**Key Points:**

- To define the role of CEM in preoperative breast cancer staging, focusing on tumour extent, detection of additional disease, and implications for surgical planning.
- CEM improves tumour extent assessment and detection of additional lesions, showing performance similar to MRI in selected clinical scenarios and influencing surgical planning in approximately 20–30% of patients.

**Graphical Abstract:**

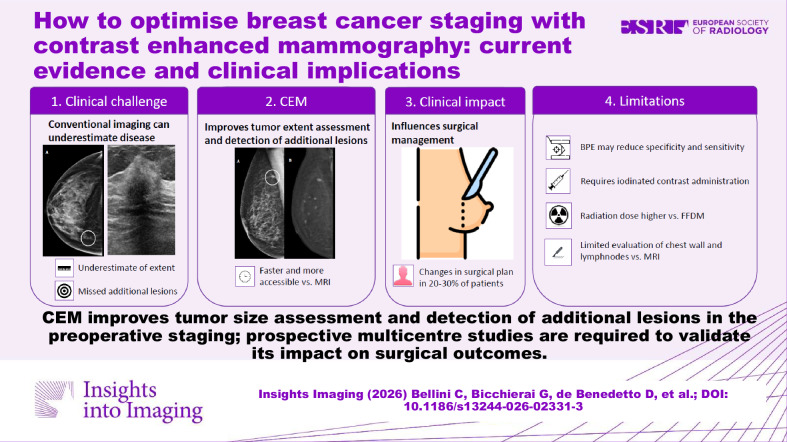

## Introduction

Accurate preoperative staging is a pivotal step in the management of breast cancer, as it directly influences surgical planning, the choice between breast conserving surgery and mastectomy, and the likelihood of achieving tumour-free margins at the first operation. Full-field digital mammography (FFDM) and ultrasound (US) remain the cornerstone of initial assessment; however, both techniques may underestimate disease extent and fail to detect additional malignant foci, particularly in dense breasts, multifocal or multicentric disease, and tumours with non-mass-forming growth patterns, potentially resulting in positive margins or re-excision [1–3].

Breast MRI is widely regarded as the most sensitive modality for local staging, because it provides a comprehensive depiction of disease extent based on tumour vascularity [4, 5]. However, MRI is not universally available, and its use may be limited by higher costs, longer examination times, contraindications, and patient intolerance [6–8]. In addition, the clinical significance of MRI-detected additional findings remains a subject of ongoing discussion, as some false-positive results may lead to additional biopsies or more extensive surgery, while the overall impact on oncological outcomes remains uncertain [9–11].

Over the past decade, contrast-enhanced mammography (CEM) has emerged as a functional breast imaging technique that combines the morphological information provided by mammography with functional information related to tumour vascularity obtained through intravenous administration of iodinated contrast media [12]. By exploiting tumour neoangiogenesis, CEM enables visualisation of iodinated contrast uptake within malignant lesions, allowing a more accurate tumour detection and extent assessment compared with conventional imaging alone [13]. Several studies have reported diagnostic performance of CEM comparable to that of breast MRI in selected clinical scenarios, including preoperative assessment of tumour size and detection of additional disease [14–16]. Shorter acquisition times, broader availability, and easier integration into routine clinical workflows represent additional features that may favour its use in the presurgical setting [17–19]. Initially adopted mainly as an alternative in patients with contraindications to MRI, CEM has progressively expanded its clinical applications [20]. Current indications include problem-solving after inconclusive conventional imaging, assessment of screening recalls, preoperative staging, and evaluation of response to neoadjuvant systemic therapy [12]. Although CEM is not yet broadly recommended in current breast cancer staging guidelines [21, 22], the European Commission Initiative on Breast Cancer suggests its use prior to surgical planning, and research interest in this setting is increasing [23].

This state-of-the-art narrative review summarises and critically discusses current evidence on the role of CEM in preoperative breast cancer staging, with attention to impact on surgical planning, performance in specific histological subtypes, and management of additional lesions, highlighting strengths and limitations relevant to daily clinical practice.

## Technical aspects of CEM

### System requirements and contrast administration

CEM is a dual-energy technique that combines conventional mammographic acquisition with functional information obtained after intravenous administration of iodinated contrast media [24]. Its biological rationale relies on tumour-induced neoangiogenesis and increased vascular permeability, resulting in preferential accumulation of iodinated contrast agent within malignant tissue [25]. This allows selective visualisation of tumour-related enhancement while suppressing background breast tissue.

After contrast injection, each mammographic view is acquired using two different X-ray spectra. Low-energy images (LE) are obtained at energy levels comparable to standard FFDM and provide morphological information, including breast density, masses, architectural distortion, and microcalcifications [26, 27]. High-energy images are acquired at higher kilovoltage levels, optimised to exploit the K-edge of iodine. Post-processing algorithms combine the low- and high-energy datasets to generate recombined images (RC), which depict iodine distribution while suppressing background breast tissue.

Standard protocols typically include bilateral craniocaudal (CC) and mediolateral oblique (MLO) views acquired within a limited time window after contrast administration, usually starting approximately 2 min after injection. Delayed acquisitions may be performed in selected cases, but current evidence does not support a consistent clinical benefit [28, 29]. There is also no consensus on the optimal acquisition sequence (order of views and which breast first), and practice varies across vendors and institutions [30]. In addition, differences in hardware, acquisition parameters, and post-processing algorithms across vendors may influence image appearance and potentially affect reproducibility and inter-study comparability.

The radiation dose of CEM is higher than that of FFDM but remains within accepted diagnostic reference levels and is generally considered acceptable for clinical use when appropriately indicated [31–34].

Non-ionic low-osmolar iodinated contrast media approved for intravenous use in computed tomography are routinely employed [35]. A commonly used protocol administers 1.5 mL/kg at ~3 mL/s using a power injector, up to a maximum of 120 mL. In some institutions, a fixed contrast dose is used instead of weight-based dosing, typically ranging between approximately 80 and 100 mL depending on local protocols and contrast agent concentration. Patient selection requires careful assessment of renal function and history of iodinated contrast allergy according to established safety guidelines [36]. Large series and systematic reviews report a low incidence of adverse reactions, mostly mild and self-limiting [30].

### Image interpretation

Image interpretation is based on integrated evaluation of LE and RC, which provide complementary morphological and functional information. LE are assessed for conventional mammographic findings, while RC are analysed for the presence, extent, and distribution of contrast enhancement. Correlation between enhancement patterns and morphological features is essential for accurate lesion detection and characterisation [16].

Background parenchymal enhancement (BPE) should be systematically assessed, as it may significantly affect lesion detectability and diagnostic performance, particularly in patients with moderate or marked enhancement. Increased BPE may obscure malignant lesions, reduce sensitivity, or mimic pathological enhancement, thereby increasing the risk of both false-negative and false-positive interpretations. Recent evidence has further highlighted that higher levels of BPE on CEM can negatively impact diagnostic accuracy, with important consequences for presurgical staging, treatment planning, and overall clinical decision-making [37]. BPE may vary with hormonal status, breast density, and acquisition timing, and contributes to inter-reader variability, similarly to breast MRI [37–39]. However, available studies suggest that the agreement between BPE assessment on MRI and CEM is only fair to moderate; in addition, technical factors, specific to mammographic acquisition, such as breast compression, may also influence BPE levels on CEM [40]. To support standardised reporting, the American College of Radiology has incorporated CEM into the BI-RADS® framework with descriptors for enhancement presence, morphology, and distribution on RC [41].

## Presurgical staging: surgical questions that imaging must answer

From a practical perspective, CEM reports in the presurgical setting should clearly describe the overall extent of disease, including the size of the index lesion, the presence and distribution of additional enhancing foci, and their spatial relationship with key anatomical structures such as the nipple–areolar complex, skin, and chest wall. When additional suspicious areas of enhancement are detected, their location and imaging characteristics should be reported to guide targeted second-look imaging and image-guided biopsy when appropriate.

### Assessment of true tumour size and extent

Accurate assessment of tumour size and extent is central to presurgical planning because it influences the likelihood of achieving negative margins at the first operation. Underestimation of tumour extent may result in incomplete excision and re-intervention, whereas overestimation may prompt unnecessarily extensive surgery. These issues are especially relevant in tumours with diffuse growth or subtle imaging manifestations, where conventional imaging may fail to capture the full disease extent. Imaging should therefore describe the overall distribution of disease within the breast, including the number of involved quadrants and the total extent of suspected or confirmed malignancy. Unifocal or multifocal disease confined to a single quadrant may still be compatible with breast-conserving surgery, whereas a large tumour-to-breast size ratio or a multicentric pattern often requires alternative surgical strategies.

In this context, CEM has primarily been explored for refining the assessment of primary tumour size [42]. Compared with FFDM and/or US, CEM generally increases sensitivity and improves delineation of the disease extent [43–47]. When compared to breast MRI, CEM has been shown to be potentially as sensitive as MRI in the evaluation of the extent of the index lesion, with a higher positive predictive value (PPV) [48–52]. Tumour size estimates obtained with CEM often show close agreement with pathological measurements; when misestimation occurs, overestimation is typically limited and broadly comparable to that reported for MRI [43, 44]. Patient- and tumour-related factors may influence accuracy, including breast density, BPE, and tumour biology [53].

Histological subtype also matters. Invasive lobular carcinoma (ILC) and ductal carcinoma in situ (DCIS), particularly low-grade forms, may show weak or heterogeneous enhancement on CEM, consistent with lower degrees of neoangiogenesis and potentially affecting delineation of tumour extent [54–56]. Nevertheless, available evidence suggests that CEM performance for ILC [55, 57, 58] and DCIS can be broadly comparable to MRI for detection and size assessment in selected studies, while remaining superior to FFDM alone for extent evaluation in these settings [59].

The potential clinical relevance of improved size assessment is reflected by reports describing modifications of surgical planning in approximately 20–30% of patients when CEM findings are considered alongside conventional imaging [59–63]. However, current evidence that these changes translate into improved surgical outcomes, such as reduced re-excision rates, improved margin status, or lower local recurrence, remains limited and is largely derived from retrospective studies. Whether these changes translate into improved surgical outcomes and justify broader implementation of CEM in the presurgical setting is the subject of ongoing prospective randomised investigations, whose results are expected in the near future [64]. An overview of published literature on the performance of CEM in the presurgical setting is shown in Table 1.

**Table 1:** Overview of key-studies on the performance of CEM in the presurgical setting

Study (year)	Design	*N*	Index lesion detection	Size assessment	Additional lesions	Change in surgical management	Comparator
Jochelson et al (2013)	Prospective	52	96% (equal to 96% of MRI)	Good in comparison to pathology (slight overestimation)	MRI depicted 94% additional malignancies vs 56% of CEM	15% (vs 21% of MRI)	MRI
Fallenberg et al (2014)	Prospective	80	100% (vs 97.4% of MRI)	Best correlation with the pathology of CEM vs MRI and FFDM	-	-	MRI/FFDM
Lobbes et al (2015)	Retrospective	87	-	Comparable to MRI	-	-	MRI
Ali-Mucheru et al (2016)	Retrospective	101	98%	-	12% additional biopsies with PPV3 of 67%	20%	-
Lee-Felker et al (2017)	Retrospective	52	95% (vs 100% of MRI)	-	100% (vs 91% of MRI)	-	MRI
Patel et al (2017)	Retrospective	88	-	Better than FFDM/US in dense and non-dense breasts	-	-	FFDM/US
Kim et al (2018)	Prospective	84	92.9%, similar to MRI	-	No differences between CEM and MRI (*p* = 0.999)	30.9%	MRI
Amato et al (2019)	Retrospective	31 (ILC)	100%	Accurate in masses, less reliable in NME	84.2%	-	-
Lorek et al (2021)	Retrospective	999	-	-	87.63% vs 38.51% FFDM	20%	FFDM
Lobbes et al (2023)	Retrospective	305 (ILC)	-	Slight overestimation of both CEM and MRI	MRI has higher sensitivity (86% vs 78%) but lower specificity (79% vs 92%) vs CEM	-	MRI
Taylor et al (2023)	Prospective	59	99%	-	CEM detected fewer additional lesions than MRI, but most were false positives (49% vs 29%)	-	MRI
Giannotti et al (2024)	Retrospective	115 (ILC)	Greater than FFDM	Greater correlation than FFDM, slight overestimation as MRI	Greater than FFDM (70% vs 20%)	-	FFDM/MRI
Wang et al (2024)	Retrospective	96	Similar to MRI (92.9% vs 93.9%)	Worse correlation with pathology than MRI, but better than FFDM	-	-	FFDM/MRI
MacCallum et al (2024)	Retrospective	202	-	-	30% with a PPV3 of 43%	21%	-
Di Grezia et al (2025)	Prospective	205	-	Near-perfect correlation of CEM measurements with pathology (Pearson’s *r* = 0.995)	13.1%	6.4%	FFDM/US
Bicchierai et al (2025)	Retrospective	991	91.8%	-	18.2% additional biopsies with PPV3 of 49.7%	22.89%	-

### Evaluation of multifocality and multicentricity

Detection of additional malignant foci within the same breast or in the contralateral breast has important implications for surgical strategy. When additional suspicious lesions are identified in the same breast, their location in relation to the index tumour and their separation distance should be clearly reported.

Comparative studies generally show higher sensitivity of MRI than CEM for detection of additional ipsilateral lesions, while CEM tends to show higher specificity and fewer false-positive findings [50, 51, 65]. This trade-off has been confirmed across studies focusing on additional tumour foci, with MRI sensitivity counterbalanced by higher false-positive rates and CEM showing a more favourable specificity profile [66, 67]. The available evidence on additional malignant findings detected with preoperative CEM is summarised in Table 1. Across published series, when additional enhancing lesions are biopsied, the reported positive predictive value for malignancy (PPV3) varies across studies, generally ranging from approximately 43% to 63% [61, 62, 68].

CEM has also demonstrated value in the detection of contralateral breast cancers, leading to modification of the surgical plan with contralateral surgery in approximately 1.4%–3.9% of patients [61, 68].

Because enhancement is not specific to malignancy, correlation with targeted second-look examinations is essential. When additional enhancing lesions are detected at CEM, management typically relies on targeted second-look ultrasound combined with retrospective review of mammography and, when available, digital breast tomosynthesis (DBT), enabling biopsy with conventional guidance in many cases [69, 70]. In a retrospective cohort of 128 patients undergoing CEM for presurgical locoregional staging, the combined use of second-look ultrasound and DBT enabled identification of a corresponding finding in 91.2% of additional malignant lesions [69].

A subset of lesions remains visible exclusively on RC, without a correlate on LE or US, with wide prevalence reported across studies up to 31.2% [71–73]. These contrast-only findings are clinically relevant because a non-negligible proportion are malignant, up to 50% in a retrospective study of 2022, supporting dedicated CEM-guided biopsy strategies when available [74]. Recognition and appropriate management of RC-only lesions are important to avoid underestimation of disease extent in CEM-based presurgical workflows. A detailed discussion of CEM-guided biopsy is beyond the scope of this review; readers are referred to a recent dedicated review addressing technical aspects and clinical implementation [71].

### Distance from the nipple–areolar complex, skin, and chest wall

Evaluation of tumour extension to the nipple–areolar complex, skin, and chest wall represents a critical component of presurgical staging, as involvement may influence both surgical planning and tumour staging. While CEM provides functional information, its ability to assess these anatomical regions remains uneven.

A structural limitation of CEM is the incomplete visualisation of the chest wall. Tumour involvement of ribs, intercostal muscles, or serratus anterior defines T4 disease according to the TNM classification; however, these structures are not reliably assessable with CEM [66]. Only the contour of the pectoralis muscle may be visible on RC, largely reflecting subtraction artefacts rather than true tissue characterisation. Consequently, suspected posterior extension or chest wall involvement should prompt complementary evaluation, most commonly targeted ultrasound and, when clinically relevant, breast MRI [17]. In addition, due to the limited field of view of mammographic acquisition, CEM does not allow evaluation of internal mammary lymph nodes, which therefore require complementary imaging when clinically relevant.

Assessment of skin and nipple–areolar complex involvement can also be challenging. Recent evidence suggests that specific CEM features, such as nipple retraction, periareolar skin thickening, disrupted superficial linear enhancement, and reduced enhancement-to-nipple distance, may be associated with pathologic nipple involvement, supporting a possible “rule-in” role in preoperative decision-making [75]. Nevertheless, subtle or early NAC involvement remains more difficult to evaluate with CEM than with MRI.

To facilitate translation of current evidence into daily clinical practice, Table 2 summarises common presurgical scenarios in which CEM may support presurgical decision-making, along with suggested management strategies.

**Table 2:** Common presurgical scenarios and suggested management.

Clinical scenario	Role of CEM	Suggested management
Newly diagnosed unifocal breast cancer with concordant FFDM and US	Assessment of tumour extent	Proceed to surgical planning if CEM findings are concordant
Suspected multifocal or multicentric disease	Detection of additional enhancing foci	Targeted second-look US and retrospective review of FFDM/DBT
An additional enhancing lesion was detected at CEM	Assessment of disease extent	Image-guided biopsy; consider CEM-guided biopsy for RC-only lesions
ILC	Consider the lower conspicuity of enhancement in comparison to NST cancers	Consider MRI if uncertainty persists
DCIS, particularly low-grade	Variable or absent enhancement	Correlate with calcifications and conventional imaging
Suspected nipple–areolar complex involvement	Possible role as a “rule-in” tool	Correlate clinically
Suspected chest wall or pectoralis muscle involvement	Incomplete assessment with CEM	Targeted ultrasound and/or MRI

Selected presurgical CEM cases are shown in Figs. 1–9.

**Fig. 1:**
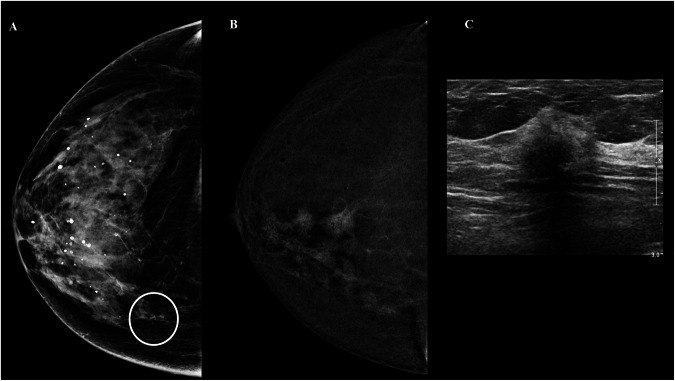
Preoperative CEM in a 70-year-old woman with dense breasts. LE CC image (**A**) shows a focal asymmetry in the upper inner quadrant of the right breast (white circle), with a corresponding small hypoechoic nodule on ultrasound of 7 mm (**C**), diagnosed as DCIS at US-guided biopsy. Recombined images (**B**) reveal extensive segmental non-mass enhancement, suggestive of more extensive disease, confirmed at final histology. Final pathology after mastectomy showed multifocal invasive ductal carcinoma NST, grade 3, measuring 2.3 mm, associated with multicentric G3 DCIS, staged pT1a, pN0(sn)

**Fig. 2:**
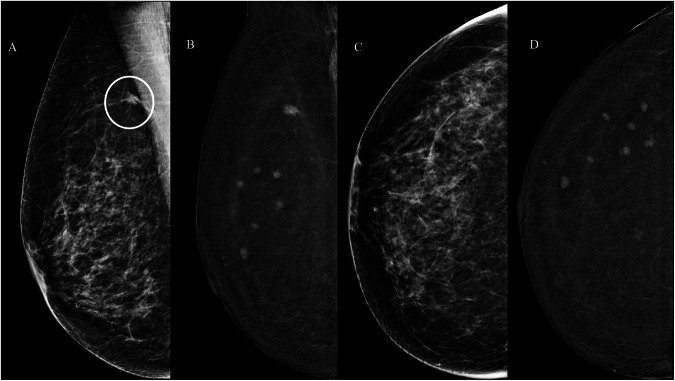
Preoperative CEM in a 59-year-old woman, with invasive carcinoma in the axillary tail of the right breast, the patient refused an MRI due to severe claustrophobia. LE MLO image (**A**) shows an irregular mass in the right axillary tail (white circle), not in the field of view in CC LE view (**C**), diagnosed as IDC at US-guided biopsy. Recombined images (**B**, **D**) reveal multiple suspicious enhancing masses, consistent with multifocal disease, which was confirmed with US-second look and at definitive histology

**Fig. 3:**
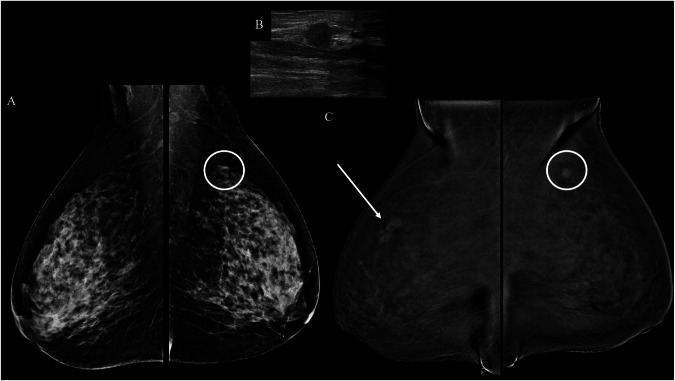
Preoperative CEM in a 61-year-old woman with dense breasts. An invasive carcinoma of the upper outer quadrant of the left breast was previously diagnosed by ultrasound-guided biopsy. LE MLO images (**A**) show an irregular mass in the left upper outer quadrant (white circle), with corresponding ultrasound findings shown in (**B**). Recombined MLO images (**C**) reveal a mass in the site of the index lesion in the upper-outer left quadrant and an additional area of heterogeneous non-mass enhancement in the upper-central region of the right breast (white arrow). The latter was negative at US-second look and showed a subtle architectural distortion on digital breast tomosynthesis (DBT), which resulted in DCIS at DBT-guided biopsy. Final pathology after bilateral surgical excision confirmed a 20-mm grade 3 DCIS in the right breast and an 8-mm grade 3 invasive papillary carcinoma (Luminal B) in the left breast

**Fig. 4:**
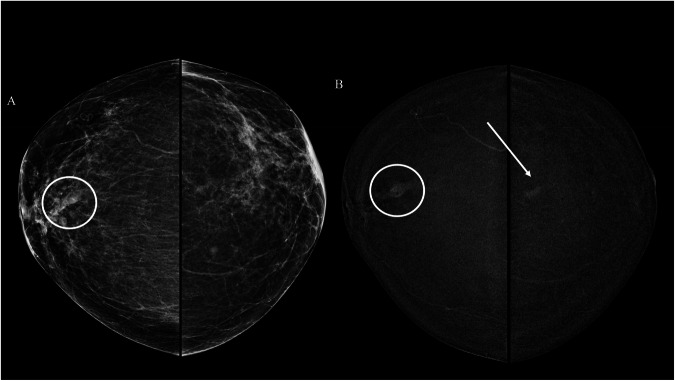
Preoperative CEM in a 61-year-old woman with non-dense breasts and a personal history of breast cancer treated with conservative surgery on the left breast. LE CC images (**A**) show an irregular mass in the central region of the right breast (white circle). Recombined MLO images (**B**) demonstrate enhancement of the index lesion in the central region of the right breast and an additional irregular enhancing mass in the central region of the left breast (white arrow). The latter showed a sonographic correlate as a small 5-mm nodule and was diagnosed as malignant at biopsy. Final histology revealed a 27-mm multifocal grade 3 invasive micropapillary carcinoma (Luminal B) in the right breast and a 5-mm grade 1 invasive cribriform carcinoma (Luminal A) in the left breast

**Fig. 5:**
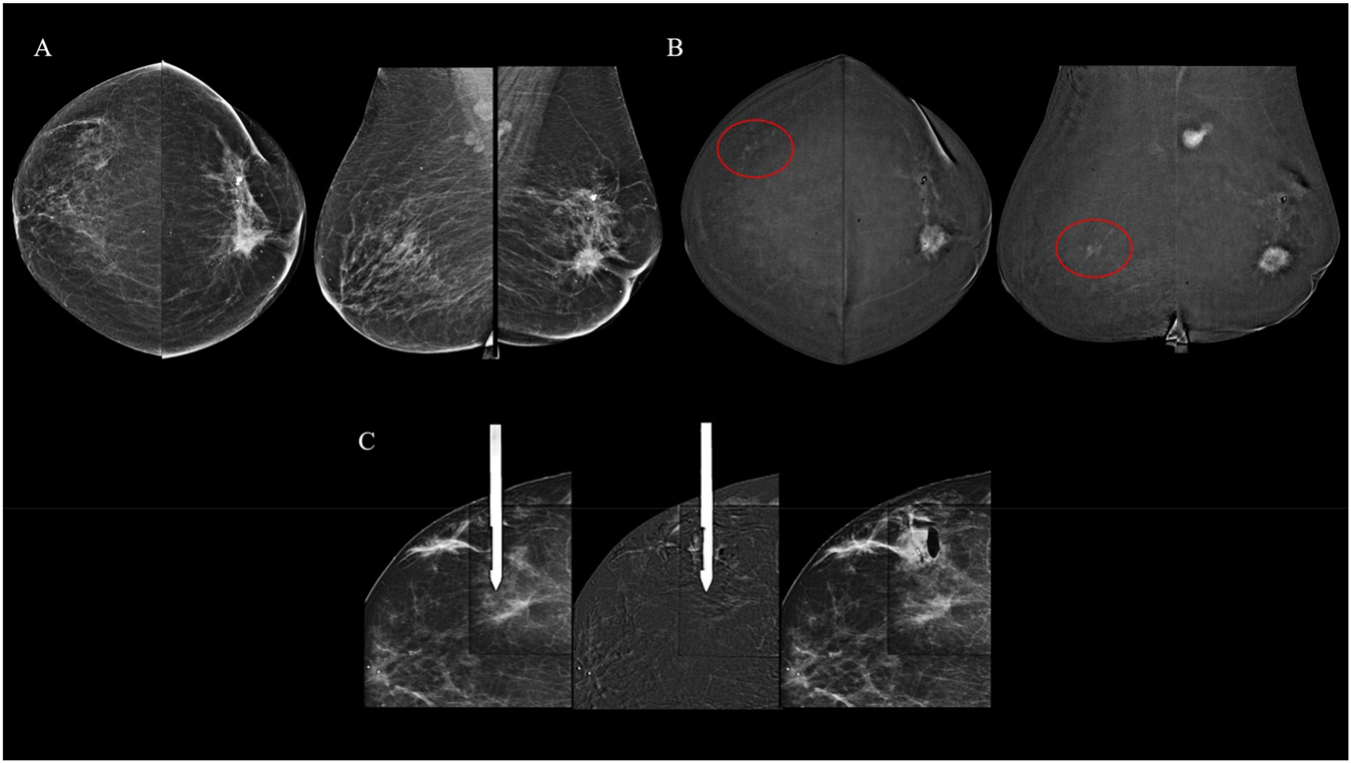
Preoperative CEM for locoregional staging in a 58-year-old woman with node-positive invasive ductal carcinoma of the left central outer quadrant. LE images (**A**) show the known index lesion in the left breast. Recombined images (**B**) demonstrate enhancement of the index lesion in the left central outer quadrant and an additional suspicious non-mass enhancement in the right upper outer quadrant (red circle). The area showed no correlation at second-look ultrasound or digital breast tomosynthesis. **C** CEM-guided biopsy of the enhancing area was therefore performed, yielding a diagnosis of DCIS

**Fig. 6:**
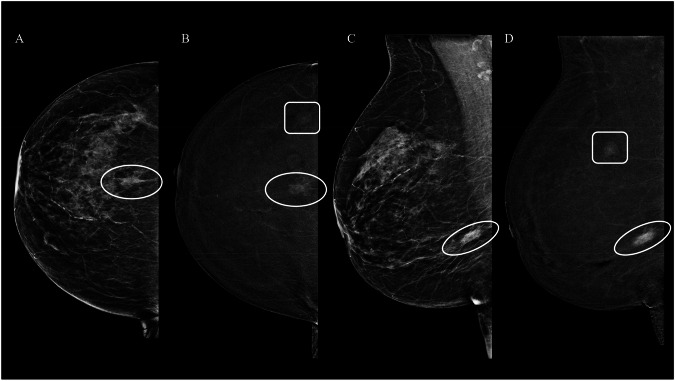
Preoperative CEM in a 70-year-old woman with non-dense breasts and a personal history of left breast cancer treated with breast-conserving surgery (left breast not shown). LE images (**A**, **C**) show a palpable irregular mass in the lower-central region of the right breast (white circle), corresponding to an invasive ductal carcinoma. Recombined images (**B**, **D**) demonstrate high-conspicuity, irregular enhancement of the index lesion. In addition, a second lesion is detected in the upper outer quadrant of the right breast (white square), showing lower conspicuity, consistent with ILC. The different enhancement characteristics reflect the distinct biological behaviour and growth patterns of the two tumour types

**Fig. 7:**
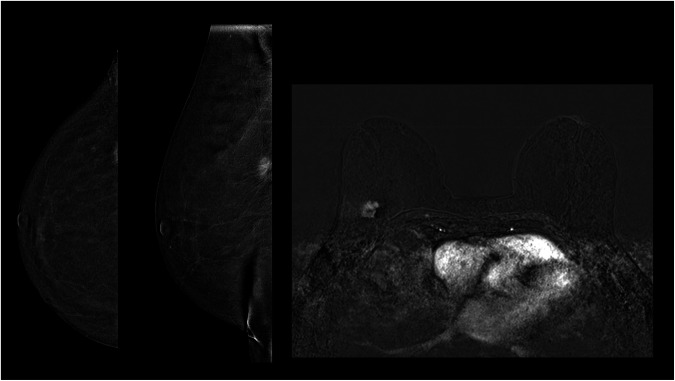
Posteriorly located breast cancer with limited assessment at CEM. CEM shows an enhancing lesion in the posterior right breast, with incomplete evaluation of the posterior extent and chest wall relationship. Subsequent breast MRI allowed more accurate assessment of tumour extent and exclusion of chest wall involvement, guiding surgical planning

**Fig. 8:**
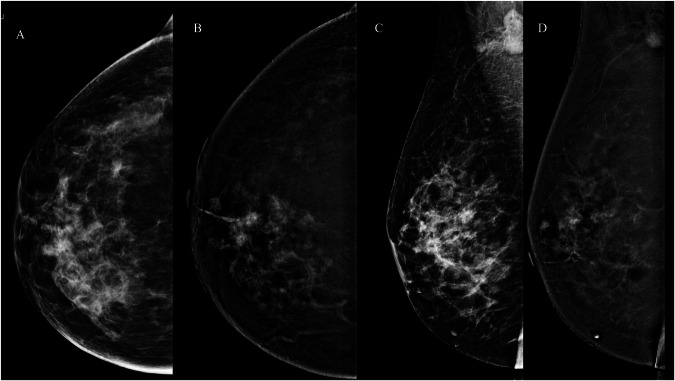
Preoperative CEM showing nipple–areolar complex involvement. LE images (**A**, **C**) demonstrate subtle architectural distortion with associated suspicious findings in the central breast. Recombined images (**B**, **D**) reveal non-mass enhancement with linear extension toward the nipple–areolar complex, raising suspicion for nipple involvement. Final histopathology confirmed tumour extension to the nipple–areolar complex

**Fig. 9:**
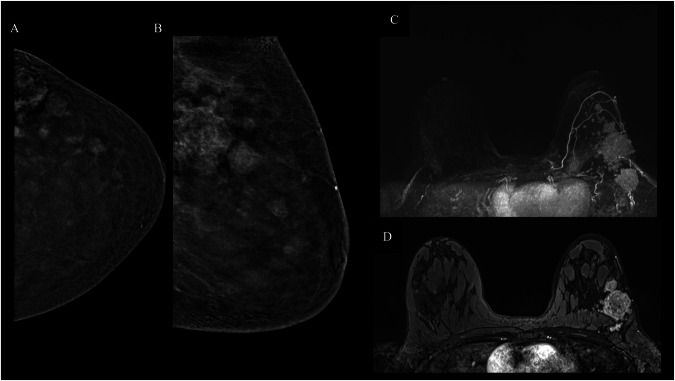
Posterior breast cancer in a patient with childhood brachial plexus palsy. The MLO view could not be optimally performed due to limited arm mobility. Recombined CEM images (**A**, **B**) show suspicious enhancement in the posterior breast. MRI maximum intensity projection (**C**) reveals an additional enhancing lesion in the axillary tail. Post-contrast non-subtracted T1-weighted MRI image (**D**) shows tumour extension with infiltration of the pectoralis muscle

## Limitations

Despite its expanding role in preoperative breast cancer staging, CEM has several limitations that should be considered in patient selection and interpretation.

CEM requires intravenous administration of iodinated contrast media, which may be contraindicated in patients with severe renal impairment or a history of severe contrast reactions. Although adverse reactions are uncommon, adherence to safety guidance and appropriate patient selection remain essential [13, 30, 35, 76, 77].

The radiation dose of CEM is higher than that of FFDM, while remaining within accepted diagnostic reference levels [31, 33, 34]. Dose considerations become particularly relevant in patients requiring repeated imaging or additional interventional procedures [78]. However, in a presurgical diagnostic setting, opposed to a screening context, this increase is generally considered justifiable when balanced against the potential clinical benefit of improved local staging [32].

From a diagnostic perspective, enhancement on CEM is not specific to malignancy. Benign lesions and BPE may lead to false-positive findings and additional work-up, even though biopsy PPV (PPV3) after abnormal CEM is comparable to MRI and higher than US in some settings [74]. Conversely, the absence of enhancement does not exclude malignancy, particularly in lesions with limited neoangiogenesis, such as low-grade DCIS. In cases of non-enhancing or weakly enhancing lesions, a structured troubleshooting approach, addressing technical factors, lesion biology, and correlation with LE images and ultrasound, may help distinguish true-negative findings from technical or biological causes of reduced enhancement (Table 3).

**Table 3:** Checklist in case of a non-enhancing lesion on RC images

Aspect to verify	Practical considerations	Tips
Adequate contrast administration	Confirm correct contrast dose, injection rate, and timing	Standard protocols typically use 1.5 mL/kg (max 120 mL) at ~3 mL/s, with acquisition starting ~2 min after injection
Contrast extravasation	Exclude partial or complete extravasation at the injection site	Assess enhancement of intramammary or subcutaneous vessels to confirm adequate contrast delivery.
Acquisition timing	Consider delayed acquisition in case of slow circulation or late enhancement	Repeat acquisition
Field of view and positioning	Ensure the lesion is fully included in the field of view	If not, consider MRI
BPE	Evaluate whether high BPE may reduce lesion conspicuity	Currently, no evidence supports menstrual cycle–based scheduling for CEM as in MRI
Tumour biology	Consider histological subtype with low neoangiogenesis (e.g. ILC, low-grade DCIS)	Integrate RC findings with LE images, especially calcifications

Another limitation of CEM compared with breast MRI is the lack of dynamic contrast enhancement analysis. While MRI allows evaluation of enhancement kinetics over time, CEM acquisitions are typically obtained at a single post-contrast time point and therefore do not provide kinetic information that may contribute to lesion characterisation.

CEM is also limited for the evaluation of posterior breast tissue and chest wall extension. Compared with MRI, CEM is less effective in assessing tumour extension to the chest wall or pectoralis muscles; when these questions are clinically relevant, MRI remains the preferred modality when available. Variability in acquisition protocols, post-processing algorithms, and reader experience may further influence performance, particularly in less standardised settings.

Breast implants have also historically been regarded as a potential limitation for CEM, mainly due to concerns related to artefacts on RC, which could impair lesion visibility. However, preliminary evidence suggests that CEM can be feasibly performed in augmented breasts using appropriate techniques, such as implant displacement views, like the Eklund manoeuvre, showing good concordance with MRI for presurgical assessment, although current evidence remains limited and derives from selected patient cohorts [79].

Finally, CEM is intrinsically limited in locoregional nodal assessment. As the technique uses the same patient positioning as conventional mammography, complete visualisation of axillary, supraclavicular, and internal mammary lymph node stations is not feasible, even with additional views. Consequently, CEM cannot provide comprehensive clinical nodal (cN) staging and does not contribute to clinical metastatic (cM) assessment. Targeted ultrasound remains essential for axillary evaluation and may reduce reliance on MRI in selected settings [17]. However, internal mammary nodes remain largely inaccessible to both techniques, and cross-sectional imaging (e.g., PET-CT) is required when advanced nodal disease is suspected or when staging is expected to change management [80].

## Future perspectives

Implementation of CEM remains heterogeneous, influenced by equipment availability, software requirements, and reimbursement frameworks [18]. Beyond these practical barriers, research is increasingly focused on whether CEM can contribute to tumour characterisation and prognostic assessment in addition to presurgical staging.

Recent studies have explored qualitative and quantitative CEM-derived biomarkers and their association with tumour biology, molecular subtypes, and prognostic factors [81, 82]. Enhancement patterns on RC have been linked to hormone receptor status, HER2 expression, and proliferative activity, while radiomics and AI-based approaches applied to both LE and RC have shown promising performance in non-invasive tumour characterisation [83–86]. In addition, growing interest has emerged in the quantitative assessment of lesion conspicuity on recombined images. A quantitative evaluation of contrast enhancement may improve the specificity of CEM in predicting malignancy by providing a more objective assessment of enhancement patterns [87]. However, these applications remain investigational. Standardisation of protocols, analysis pipelines and multicenter validation will be essential to determine whether CEM-derived biomarkers can reliably inform clinical decisions.

In parallel, emerging AI-based reconstruction strategies, such as generative adversarial networks, have been investigated in breast MRI to enable diagnostic imaging with reduced contrast agent dose [88, 89]. Similar approaches could be explored in CEM to optimise contrast utilisation, particularly in selected patients requiring repeated examinations or with relative contraindications to iodinated contrast agents.

Prospective multicentric studies are also needed to clarify whether CEM-based staging and quantitative analysis can predict clinically relevant outcomes, such as re-excision rates and short-term local recurrence, thereby defining the true prognostic value of CEM within presurgical workflows.

## Conclusions

CEM has emerged as a valuable adjunct for presurgical breast cancer staging, improving assessment of tumour extent and additional disease beyond conventional imaging. Its role is most relevant when MRI is unavailable, contraindicated, or unlikely to provide substantial incremental benefit. Current evidence supports technical feasibility and clinical utility, with optimal performance, even if influenced by tumour biology, acquisition protocols, and reader experience. Further prospective and multicentric studies are needed to define the impact of CEM on surgical outcomes and to clarify its role within personalised breast cancer management.

## Data Availability

Not applicable. No new datasets were generated or analysed for this review. All data discussed are derived from published literature.

## Abbreviations

AI: Artificial intelligence
BPE: Background parenchymal enhancement
BI-RADS®: Breast imaging reporting and data system
CC: Craniocaudal
CEM: Contrast-enhanced mammography
DBT: Digital breast tomosynthesis
DCIS: Ductal carcinoma in Situ
FFDM: Full-field digital mammography
IDC: Invasive ductal carcinoma
ILC: Invasive lobular carcinoma
LE: Low-energy images
MLO: Mediolateral oblique
MRI: Magnetic resonance imaging
NAC: Nipple–areolar complex
NST: No special type
PET/CT: Positron emission tomography/computed tomography
PPV: Positive predictive value
PPV3: Positive predictive value of biopsy
RC: Recombined images
TNM: Tumour–node–metastasis
US: Ultrasound

## References

[1] Kuhl C, Kuhn W, Braun M, Schild H (2007) Pre-operative staging of breast cancer with breast MRI: One step forward, two steps back? Breast 16:S34–S44 17959382 10.1016/j.breast.2007.07.014

[2] Dummin LJ, Cox M, Plant L (2007) Prediction of breast tumor size by mammography and sonography—a breast screen experience. Breast 16:38–46 16846736 10.1016/j.breast.2006.04.003

[3] Heusinger K, Löhberg C, Lux MP et al (2005) Assessment of breast cancer tumor size depends on method, histopathology and tumor size itself. Breast Cancer Res Treat 94:17–23 16142441 10.1007/s10549-005-6653-x

[4] Mann RM, Kuhl CK, Kinkel K, Boetes C (2008) Breast MRI: guidelines from the European Society of Breast Imaging. Eur Radiol 18:1307–1318 18389253 10.1007/s00330-008-0863-7 PMC2441490

[5] Sardanelli F, Trimboli RM, Houssami N et al (2022) Magnetic resonance imaging before breast cancer surgery: results of an observational multicenter international prospective analysis (MIPA). Eur Radiol 32:1611–1623 34643778 10.1007/s00330-021-08240-x PMC8831264

[6] Sardanelli F, Magni V, Rossini G, Kilburn-Toppin F, Healy NA, Gilbert FJ (2024) The paradox of MRI for breast cancer screening: high-risk and dense breasts-available evidence and current practice. Insights Imaging 15:96 38536530 10.1186/s13244-024-01653-4 PMC10973307

[7] Clauser P, Mann R, Athanasiou A et al (2018) A survey by the European Society of Breast Imaging on the utilisation of breast MRI in clinical practice. Eur Radiol 28:1909–1918 29168005 10.1007/s00330-017-5121-4 PMC5882636

[8] Berg WA, Bandos AI, Sava MG (2023) Analytic hierarchy process analysis of patient preferences for contrast-enhanced mammography versus MRI as supplemental screening options for breast cancer. J Am Coll Radiol 20:758–768 37394083 10.1016/j.jacr.2023.05.014

[9] Arnaout A, Catley C, Booth CM et al (2015) Use of preoperative magnetic resonance imaging for breast cancer: a Canadian population-based study. JAMA Oncol 1:1238–1250 26402040 10.1001/jamaoncol.2015.3018

[10] Jatoi I, Benson JR (2013) The case against routine preoperative breast MRI. Future Oncol 9:347–353 23469970 10.2217/fon.12.186

[11] Zeng Z, Amin A, Roy A et al (2020) Preoperative magnetic resonance imaging use and oncologic outcomes in premenopausal breast cancer patients. npj Breast Cancer 6:49 33083528 10.1038/s41523-020-00192-7 PMC7532157

[12] Schiaffino S, Cozzi A, Clauser P et al (2024) Current use and future perspectives of contrast-enhanced mammography (CEM): a survey by the European Society of Breast Imaging (EUSOBI). Eur Radiol 34:5439–5450 38227202 10.1007/s00330-023-10574-7

[13] Jochelson MS, Lobbes MBI (2021) Contrast-enhanced mammography: state of the art. Radiology 299:36–48 33650905 10.1148/radiol.2021201948 PMC7997616

[14] Neeter LMFH, Robbe MMQ, van Nijnatten TJA et al (2023) Comparing the diagnostic performance of contrast-enhanced mammography and breast MRI: a systematic review and meta-analysis. J Cancer 14:174–182 36605487 10.7150/jca.79747 PMC9809339

[15] Gelardi F, Ragaini EM, Sollini M, Bernardi D, Chiti A (2022) Contrast-enhanced mammography versus breast magnetic resonance imaging: a systematic review and meta-analysis. Diagnostics (Basel) 12:1890 36010240 10.3390/diagnostics12081890 PMC9406751

[16] Cozzi A, Magni V, Zanardo M, Schiaffino S, Sardanelli F (2022) Contrast-enhanced mammography: a systematic review and meta-analysis of diagnostic performance. Radiology 302:568–581 34904875 10.1148/radiol.211412

[17] Lobbes MBI, Heuts EM, Moossdorff M, van Nijnatten TJA (2021) Contrast enhanced mammography (CEM) versus magnetic resonance imaging (MRI) for staging of breast cancer: the pro CEM perspective. Eur J Radiol 142:109883 34358810 10.1016/j.ejrad.2021.109883

[18] Sáenz JA, Baltzer PAT, Allajbeu I et al (2025) Overview of different reimbursement strategies among contrast-enhanced mammography (CEM) expert centers on a global level—a survey study. Eur J Radiol 191:112315 40682981 10.1016/j.ejrad.2025.112315

[19] Pires-Gonçalves L, Henriques Abreu M, Ferrão A et al (2023) Patient perspectives on repeated contrast-enhanced mammography and magnetic resonance during neoadjuvant chemotherapy of breast cancer. Acta Radiol 64:1816–1822 36575580 10.1177/02841851221144021

[20] Sardanelli F, Fallenberg EM, Clauser P et al (2017) Mammography: an update of the EUSOBI recommendations on information for women. Insights Imaging 8:11–18 27854006 10.1007/s13244-016-0531-4 PMC5265195

[21] Bevers TB, Helvie M, Bonaccio E et al (2018) Breast cancer screening and diagnosis, version 3.2018, NCCN clinical practice guidelines in oncology. J Natl Compr Canc Netw 16:1362–1389 30442736 10.6004/jnccn.2018.0083

[22] National Comprehensive Cancer Network (2026) NCCN clinical practice guidelines in oncology: breast cancer. Available via https://www.nccn.org/guidelines/guidelines-detail?category=2&id=1421. Accessed 22 Jan 2026

[23] European Commission Initiative on Breast Cancer (2026) Cancer screening, diagnosis and care. Available via https://cancer-screening-and-care.jrc.ec.europa.eu/en/ecibc. Accessed 22 Jan 2026

[24] Dromain C, Balleyguier C, Adler G, Garbay JR, Delaloge S (2009) Contrast-enhanced digital mammography. Eur J Radiol 69:34–42 18790584 10.1016/j.ejrad.2008.07.035

[25] Weidner N, Semple JP, Welch WR, Folkman J (1991) Tumor angiogenesis and metastasis–correlation in invasive breast carcinoma. N Engl J Med 324:1–8 1701519 10.1056/NEJM199101033240101

[26] Francescone MA, Jochelson MS, Dershaw DD et al (2014) Low energy mammogram obtained in contrast-enhanced digital mammography (CEDM) is comparable to routine full-field digital mammography (FFDM). Eur J Radiol 83:1350–1355 24932846 10.1016/j.ejrad.2014.05.015

[27] Lalji UC, Jeukens CRLPN, Houben I et al (2015) Evaluation of low-energy contrast-enhanced spectral mammography images by comparing them to full-field digital mammography using EUREF image quality criteria. Eur Radiol 25:2813–2820 25813015 10.1007/s00330-015-3695-2 PMC4562003

[28] Calabrò N, Abruzzese F, Valentini E et al (2024) Evaluating the impact of delayed-phase imaging in contrast-enhanced mammography on breast cancer staging: a comparative study of abbreviated versus complete protocol. Radiol Med 129:989–998 38987501 10.1007/s11547-024-01838-3 PMC11252175

[29] Xu W, Zheng B, Chen W et al (2021) Can the delayed phase of quantitative contrast-enhanced mammography improve the diagnostic performance on breast masses? Quant Imaging Med Surg 11:3684–3697 34341742 10.21037/qims-20-1092 PMC8245946

[30] Zanardo M, Cozzi A, Trimboli RM et al (2019) Technique, protocols and adverse reactions for contrast-enhanced spectral mammography (CESM): a systematic review. Insights Imaging 10:76 31376021 10.1186/s13244-019-0756-0 PMC6677840

[31] Bicchierai G, Busoni S, Tortoli P et al (2022) Single center evaluation of comparative breast radiation dose of contrast enhanced digital mammography (CEDM), digital mammography (DM) and digital breast tomosynthesis (DBT). Acad Radiol 29:1342–1349 35065889 10.1016/j.acra.2021.12.022

[32] Phillips J, Mihai G, Hassonjee SE et al (2018) Comparative dose of contrast-enhanced spectral mammography (CESM), digital mammography, and digital breast tomosynthesis. AJR Am J Roentgenol 211:839–846 30063367 10.2214/AJR.17.19036

[33] Jeukens CRLPN, Lalji UC, Meijer E et al (2014) Radiation exposure of contrast-enhanced spectral mammography compared with full-field digital mammography. Invest Radiol 49:659–665 24872005 10.1097/RLI.0000000000000068

[34] James JR, Pavlicek W, Hanson JA et al (2017) Breast radiation dose with CESM compared with 2D FFDM and 3D tomosynthesis mammography. AJR Am J Roentgenol 208:362–372 28112559 10.2214/AJR.16.16743

[35] van Nijnatten TJA, Meltem E, van der Molen AJ et al (2026) Contrast-associated risks of iodine-based contrast media administration in breast imaging: tips and overview of existing evidence—a narrative review. Eur J Radiol 194:112481 41138316 10.1016/j.ejrad.2025.112481

[36] American College of Radiology (2026) ACR manual on contrast media. Available via https://www.acr.org/Clinical-Resources/Clinical-Tools-and-Reference/Contrast-Manual. Accessed 20 Jan 2026

[37] Bechyna S, Santonocito A, Pötsch N et al (2025) Impact of background parenchymal enhancement (BPE) on diagnostic performance of contrast-enhanced mammography (CEM) for breast cancer diagnosis. Eur J Radiol 188:112145 40318502 10.1016/j.ejrad.2025.112145

[38] Moffa G, Galati F, Spagnoli A et al (2025) BPE on contrast-enhanced mammography: relationship with breast density, age and menopausal status. Radiol Med 130:74–80 39535654 10.1007/s11547-024-01912-w

[39] Magni V, Cozzi A, Muscogiuri G et al (2024) Background parenchymal enhancement on contrast-enhanced mammography: associations with breast density and patient’s characteristics. Radiol Med 129:1303–1312 39060886 10.1007/s11547-024-01860-5

[40] Ferrara F, Santonocito A, Vogel W et al (2025) Background parenchymal enhancement in CEM and MRI: Is there always a high agreement? Eur J Radiol 183:111903 39736216 10.1016/j.ejrad.2024.111903

[41] American College of Radiology (2026) ACR breast imaging reporting & data system (BI-RADS®). Available via https://www.acr.org/Clinical-Resources/Clinical-Tools-and-Reference/Reporting-and-Data-Systems/BI-RADS. Accessed 22 Jan 2026

[42] Tsarouchi M, Hoxhaj A, Portaluri A et al (2025) Breast cancer staging with contrast-enhanced imaging. The benefits and drawbacks of MRI, CEM, and dedicated breast CT. Eur J Radiol 185:112013 40036929 10.1016/j.ejrad.2025.112013

[43] Łuczyńska E, Niemiec J, Hendrick E et al (2016) Degree of enhancement on contrast enhanced spectral mammography (CESM) and lesion type on mammography (MG): comparison based on histological results. Med Sci Monit 22:3886–3893 27768681 10.12659/MSM.900371 PMC5077289

[44] Patel BK, Garza SA, Eversman S et al (2017) Assessing tumor extent on contrast-enhanced spectral mammography versus full-field digital mammography and ultrasound. Clin Imaging 46:78–84 28750354 10.1016/j.clinimag.2017.07.001

[45] Sogani J, Mango VL, Keating D et al (2021) Contrast-enhanced mammography: past, present, and future. Clin Imaging 69:269–279 33032103 10.1016/j.clinimag.2020.09.003 PMC8494428

[46] Di Grezia G, Mercogliano S, Marinelli L et al (2025) Contrast-enhanced mammography in breast lesion assessment: accuracy and surgical impact. Tomography 11:93 40863884 10.3390/tomography11080093 PMC12389778

[47] Åhsberg K, Gardfjell A, Nimeus E et al (2020) Added value of contrast-enhanced mammography (CEM) in staging of malignant breast lesions—a feasibility study. World J Surg Oncol 18:100 32438917 10.1186/s12957-020-01865-0 PMC7243325

[48] Lee-Felker SA, Tekchandani L, Thomas M et al (2017) Newly diagnosed breast cancer: comparison of contrast-enhanced spectral mammography and breast MR imaging in the evaluation of extent of disease. Radiology 285:389–400 28654337 10.1148/radiol.2017161592

[49] Lobbes MBI, Lalji UC, Nelemans PJ et al (2015) The quality of tumor size assessment by contrast-enhanced spectral mammography and the benefit of additional breast MRI. J Cancer 6:144–150 25561979 10.7150/jca.10705 PMC4280397

[50] Jochelson MS, Dershaw DD, Sung JS et al (2013) Bilateral contrast-enhanced dual-energy digital mammography: feasibility and comparison with conventional digital mammography and MR imaging in women with known breast carcinoma. Radiology 266:743–751 23220903 10.1148/radiol.12121084 PMC5673037

[51] Fallenberg EM, Dromain C, Diekmann F et al (2014) Contrast-enhanced spectral mammography versus MRI: initial results in the detection of breast cancer and assessment of tumour size. Eur Radiol 24:256–264 24048724 10.1007/s00330-013-3007-7

[52] Nicosia L, Bozzini AC, Palma S et al (2022) Contrast-enhanced spectral mammography and tumor size assessment: a valuable tool for appropriate surgical management of breast lesions. Radiol Med 127:1228–1234 36149581 10.1007/s11547-022-01561-x

[53] Travieso-Aja MDM, Naranjo-Santana P, Fernández-Ruiz C et al (2018) Factors affecting the precision of lesion sizing with contrast-enhanced spectral mammography. Clin Radiol 73:296–303 29221721 10.1016/j.crad.2017.10.017

[54] van Nijnatten TJ, Jochelson MS, Pinker K et al (2019) Differences in degree of lesion enhancement on CEM between ILC and IDC. BJR Open 1:20180046 33178931 10.1259/bjro.20180046 PMC7592434

[55] Lobbes MBI, Neeter LMFH, Raat F et al (2023) The performance of contrast-enhanced mammography and breast MRI in local preoperative staging of invasive lobular breast cancer. Eur J Radiol 164:110881 37201248 10.1016/j.ejrad.2023.110881

[56] Marzogi A, Baltzer PAT, Kapetas P et al (2023) Is the level of contrast enhancement on contrast-enhanced mammography (CEM) associated with the presence and biological aggressiveness of breast cancer? Diagnostics 13:754 36832242 10.3390/diagnostics13040754 PMC9955826

[57] Giannotti E, Van Nijnatten TJA, Chen Y et al (2024) The role of contrast-enhanced mammography in the preoperative evaluation of invasive lobular carcinoma of the breast. Clin Radiol 79:e799–e806 38383254 10.1016/j.crad.2024.01.035

[58] Amato F, Bicchierai G, Cirone D et al (2019) Preoperative loco-regional staging of invasive lobular carcinoma with contrast-enhanced digital mammography (CEDM). Radiol Med 124:1229–1237 31773458 10.1007/s11547-019-01116-7

[59] Wang L, Wang P, Shao H et al (2024) Role of contrast-enhanced mammography in the preoperative detection of ductal carcinoma in situ of the breasts: a comparison with low-energy image and magnetic resonance imaging. Eur Radiol 34:3342–3351 37853174 10.1007/s00330-023-10312-z

[60] Bicchierai G, Tonelli P, Piacenti A et al (2020) Evaluation of contrast-enhanced digital mammography (CEDM) in the preoperative staging of breast cancer: large-scale single-center experience. Breast J 26:1276–1283 31999029 10.1111/tbj.13766

[61] Bicchierai G, Migliaro G, Pugliese F et al (2025) Evaluation of contrast-enhanced mammography (CEM) in the preoperative staging of breast cancer: large-scale single center experience, update to 1005 cases. Radiol Med 130:830–843 40153207 10.1007/s11547-025-02009-8

[62] Ali-Mucheru M, Pockaj B, Patel B et al (2016) Contrast-enhanced digital mammography in the surgical management of breast cancer. Ann Surg Oncol 23:649–655 27638679 10.1245/s10434-016-5567-7

[63] Lorek A, Steinhof-Radwańska K, Barczyk-Gutkowska A et al (2021) The usefulness of spectral mammography in surgical planning of breast cancer treatment-analysis of 999 patients with primary operable breast cancer. Curr Oncol 28:2548–2559 34287253 10.3390/curroncol28040232 PMC8293137

[64] Åhsberg K, Gardfjell A, Nimeus E, Ryden L, Zackrisson S (2021) The PROCEM study protocol: Added value of preoperative contrast-enhanced mammography in staging of malignant breast lesions - a prospective randomized multicenter study. BMC Cancer 21:1115 34663236 10.1186/s12885-021-08832-2 PMC8521511

[65] Kim EY, Youn I, Lee KH et al (2018) Diagnostic value of contrast-enhanced digital mammography versus contrast-enhanced magnetic resonance imaging for the preoperative evaluation of breast cancer. J Breast Cancer 21:453–462 30607168 10.4048/jbc.2018.21.e62 PMC6310721

[66] Taylor DB, Burrows S, Saunders CM, Parizel PM, Ives A (2023) Contrast-enhanced mammography (CEM) versus MRI for breast cancer staging: detection of additional malignant lesions not seen on conventional imaging. Eur Radiol Exp 7:8 36781808 10.1186/s41747-022-00318-5 PMC9925630

[67] Hafez MAF, Zeinhom A, Hamed DAA, Ghaly GRM, Tadros SFK (2023) Contrast-enhanced mammography versus breast MRI in the assessment of multifocal and multicentric breast cancer: a retrospective study. Acta Radiol 64:2868–2880 37674355 10.1177/02841851231198346

[68] MacCallum C, Elder K, Nickson C et al (2024) Contrast-enhanced mammography in local staging of screen-detected breast cancer. Ann Surg Oncol 31:6820–6830 39048901 10.1245/s10434-024-15848-y

[69] Bellini C, Bicchierai G, Amato F et al (2022) Comparison between second-look ultrasound and second-look digital breast tomosynthesis in the detection of additional lesions with presurgical CESM. Br J Radiol 95:20210927 35451312 10.1259/bjr.20210927 PMC10996408

[70] Coffey K, Sung J, Comstock C et al (2021) Utility of targeted ultrasound to predict malignancy among lesions detected on contrast-enhanced digital mammography. AJR Am J Roentgenol 217:595–604 33025811 10.2214/AJR.20.24368 PMC9295859

[71] Alcantara R, Azcona J, Pitarch M, Vall E, Vila-Trias E, Arenas EN (2025) Contrast-enhanced mammography-guided biopsy: principles, challenges, and opportunities. Insights Imaging 16:262 41284217 10.1186/s13244-025-02148-6 PMC12644378

[72] Houben IPL, de Voorde PV, Jeukens CRLPN et al (2017) Contrast-enhanced spectral mammography as work-up tool in patients recalled from breast cancer screening has low risks and might hold clinical benefits. Eur J Radiol 94:31–37 28941757 10.1016/j.ejrad.2017.07.004

[73] Viggiano T, Scott R, Sharpe R et al (2022) Contrast enhanced mammography in routine clinical practice: frequency and malignancy rates of enhancing otherwise occult findings. Clin Breast Cancer 22:e736–e744 35977855 10.1016/j.clbc.2022.07.008

[74] Amir T, Hogan MP, Jacobs S, Sevilimedu V, Sung J, Jochelson MS (2022) Comparison of false-positive versus true-positive findings on contrast-enhanced digital mammography. AJR Am J Roentgenol 218:797–808 34817195 10.2214/AJR.21.26847 PMC9110098

[75] Cozzi A, Bellini C, Girometti R et al (2025) Tumor involvement of the nipple at preoperative contrast-enhanced mammography: a multicenter diagnostic accuracy study. Radiology 317:e251350 41186466 10.1148/radiol.251350

[76] Filippone F, Boudagga Z, Frattini F et al (2024) Contrast enhancement in breast cancer: magnetic resonance vs. mammography: a 10-year systematic review. Diagnostics (Basel) 14:2400 39518367 10.3390/diagnostics14212400 PMC11545212

[77] Endrikat J, Khater H, Boreham AD et al (2023) Iopromide for contrast-enhanced mammography: a systemic review and meta-analysis of pertinent literature. Breast Cancer (Auckl) 17:11782234231189467 37600467 10.1177/11782234231189467 PMC10433886

[78] Alcantara R, Azcona J, Pitarch M et al (2025) Breast radiation dose with contrast-enhanced mammography-guided biopsy: a retrospective comparison with stereotactic and tomosynthesis guidance. Eur Radiol 35:2119–2129 39143245 10.1007/s00330-024-10920-3 PMC11914308

[79] Carnahan MB, Pockaj B, Pizzitola V et al (2021) Contrast-enhanced mammography for newly diagnosed breast cancer in women with breast augmentation: preliminary findings. AJR Am J Roentgenol 217:855–856 33728971 10.2214/AJR.20.25341

[80] Vaz SC, Woll JPP, Cardoso F et al (2024) Joint EANM-SNMMI guideline on the role of 2-[18 F]FDG PET/CT in no special type breast cancer: (endorsed by the ACR, ESSO, ESTRO, EUSOBI/ESR, and EUSOMA). Eur J Nucl Med Mol Imaging 51:2706–2732 38740576 10.1007/s00259-024-06696-9 PMC11224102

[81] Luczynska E, Piegza T, Szpor J et al (2022) Contrast-enhanced mammography (CEM) capability to distinguish molecular breast cancer subtypes. Biomedicines 10:2384 36289645 10.3390/biomedicines10102384 PMC9598186

[82] Liu Y, Zhao S, Huang J et al (2020) Quantitative analysis of enhancement intensity and patterns on contrast-enhanced spectral mammography. Sci Rep 10:9807 32555338 10.1038/s41598-020-66501-z PMC7299980

[83] Wang S, Wang Z, Li R et al (2022) Association between quantitative and qualitative image features of contrast-enhanced mammography and molecular subtypes of breast cancer. Quant Imaging Med Surg 12:1270–1280 35111622 10.21037/qims-21-589 PMC8739155

[84] Marino MA, Leithner D, Sung J et al (2020) Radiomics for tumor characterization in breast cancer patients: a feasibility study comparing contrast-enhanced mammography and magnetic resonance imaging. Diagnostics (Basel) 10:492 32708512 10.3390/diagnostics10070492 PMC7400681

[85] La Forgia D, Fanizzi A, Campobasso F et al (2020) Radiomic analysis in contrast-enhanced spectral mammography for predicting breast cancer histological outcome. Diagnostics (Basel) 10:708 32957690 10.3390/diagnostics10090708 PMC7555402

[86] Dominique C, Callonnec F, Berghian A et al (2022) Deep learning analysis of contrast-enhanced spectral mammography to determine histoprognostic factors of malignant breast tumours. Eur Radiol 32:4834–4844 35094119 10.1007/s00330-022-08538-4 PMC8800426

[87] Allajbeu I, Nanaa M, Manavaki R et al (2025) Improving the diagnostic performance of contrast-enhanced mammography through lesion conspicuity and enhancement quantification. Eur Radiol 35:6385–6397 40180638 10.1007/s00330-025-11501-8 PMC12417241

[88] Müller-Franzes G, Huck L, Tayebi Arasteh S et al (2023) Using machine learning to reduce the need for contrast agents in breast MRI through synthetic images. Radiology 307:e222211 36943080 10.1148/radiol.222211

[89] Bahl M (2023) The quest to reduce the use of gadolinium-based contrast agents: AI may provide a solution. Radiology 307:e230325 36943082 10.1148/radiol.230325 PMC10140636

